# Inhibition kinetics, molecular docking, and stability studies of the effect of papain-generated peptides from palm kernel cake proteins on angiotensin-converting enzyme (ACE)

**DOI:** 10.1016/j.fochms.2022.100147

**Published:** 2022-11-17

**Authors:** Mohammad Zarei, Raheleh Ghanbari, Najib Zainal, Reza Ovissipour, Nazamid Saari

**Affiliations:** a Future Foods Lab and Cellular Agriculture Initiative, Virginia Seafood Agricultural Research and Extension Center, Virginia Polytechnic Institute and State University, Hampton, VA 23699, USA; b Department of Food Science, Faculty of Food Science and Technology, Universiti Putra Malaysia, UPM, Serdang, Selangor 43400, Malaysia

**Keywords:** Palm kernel cake, Kinetics, Molecular docking, Angiotensin-converting enzyme inhibitory peptide

## Abstract

Three novel peptide sequences YGIKVGYAIP, GGIF, and GIFE from papain-generated protein hydrolysate of palm kernel cake proteins were used for stability study against ACE, ACE-inhibition kinetics, and molecular docking studies. Results showed that peptide YGIKVGYAIP was degraded, and its ACE-inhibitory activity decreased after 3 h pre-incubation with ACE, while peptides GGIF and GIFE were resistant. However, although the ACE-inhibitory activity of GIFE increased during this time, the ACE inhibitory activity of GGIF decreased after pre-incubation with ACE, indicating that peptide. YGIKVGYAIP and GGIF are substrate-type, whereas GIFE is a true-inhibitor type. Peptide YGIKVGYAIP showed the lowest K_i_ (0.054 mM) in the inhibition kinetics study compared to GGIF and GIFE, with K_i_ of 1.27 m M and 18 mM, respectively. In addition, YGIKVGYAIP revealed the lowest K_m_ and V_max_ and higher CE in different peptide concentrations, implying that the enzyme catalysis decreased, and peptides had some binding affinity to the enzyme in lower concentrations, which led to reduced catalytic ability. Furthermore, YGIKVGYAIP showed the lowest docking score of −14.733 and 21 interactions with tACE, while GGIF revealed the higher docking score of −8.006 with 15 interactions with tACE.

## Highlights

• Inhibition mode of the peptides at different concentrations was mixed-type inhibition.
• YGIKVGYAIP was degraded after incubation with ACE, while others were resistant and not degraded.
• Results exhibited that GIFE was a prodrug inhibitor, whereas others were true inhibitors.
• YGIKVGYAIP exhibited a higher number of total interactions according to the molecular docking study.

## 1 Introduction

Bioactive peptides with amino acid sequences between 2 and 12 are of interest to many functional food-related industries, as they may exert various biological activities. Many peptides that are derived or generated from food protein sources and are essentially composed of l-amino acid residues are the targets of functional food manufacturers, as they are a valuable source of protein that also has physiological functions such as ACE inhibitory activity (Corrons et al., 2017, Girgih et al., 2014, Hanafi et al., 2018, Muhammad Auwal et al., 2017, Samarakoon et al., 2013), DPPH• radical scavenging activity (Wang et al., 2014, Zheng et al., 2015, Zheng et al., 2018), and metal chelating activity (Intarasirisawat et al., 2013, Torres-Fuentes et al., 2012). Of these, bioactive peptides that can influence the renin-angiotensin system, thus reducing or modulating blood pressure, have received special interest due to their therapeutic potential.

ACE inhibitory peptides have been successfully generated and isolated from food protein sources such as soy (Wu & Ding, 2001), milk (FitzGerald et al., 2004), maize (Yano et al., 1996), sunflower (Megías et al., 2009), and wheat germ (Qu et al., 2012). Palm kernel cake (PKC) is another potentially valuable protein source for the production of bioactive peptides with high protein content ranging from 14.5 % to 19.5 % (Akpanabiatu et al., 2001, Lateef et al., 2008, Onwudike, 1986). It is considered an inexpensive and abundantly available by-product of oil mills in Malaysia, with an annual production of 2.5 million tons (MPOB, 2015), thus generating approximately 460,000 tons of PKC protein per year.

Recently, PKC protein hydrolysates and peptides have been shown to exhibit exceptionally strong angiotensin-converting enzyme (ACE) inhibitory and antioxidant activities (Auwal et al., 2017, Ng et al., 2013, Zarei et al., 2014, Zarei et al., 2015, Zarei et al., 2016). PKC protein hydrolysates and peptides have also been shown to reduce blood pressure in White Dawley rats (Zarei et al., 2015). However, such peptides exhibit inherent instability in the lumen of the gastrointestinal tract upon consumption as a functional food. Hence, their effectiveness against ACE has been disputed, as it either reduces or increases the peptide’s effectiveness as an inhibitor against ACE by partially degrading the molecules. There are no reports on the stability and inhibition kinetics of the bioactive peptides from palm kernel cake protein against ACE. Information about the stability and the kinetic study of a particular peptide can be used to deduce the optimal dose of the peptide required for its desired effect. (Girgih et al., 2014). Therefore, this study aimed to determine the effect of ACE on the degradation of the generated peptides as well as the inhibition kinetics of ACE as affected by peptides generated from PKC proteins and their ACE-peptide binding mechanisms.

## 2 Material and methods

### 2.1 Materials

PKC was obtained from the My-4-Season company, Serdang, Malaysia. Hippuryl-l-Histidyl-l-Leucine (HHL), the angiotensin-I-converting enzyme from rabbit lung (2 units/mg protein) with CAS:9015-82-1 and captopril were purchased from Sigma Chemical Co. (St. Louis, MO, USA). Sodium chloride, boric acid and sodium borate were purchased from Merck Co. (Darmstadt, Germany). Pyridine, benzene sulfonyl chloride (BSC), and hydrochloric acid were obtained from Fisher Scientific (Georgia, USA).

### 2.2 Production and identification of bioactive peptides

Protein hydrolysis and identification of bioactive peptides were carried out following the methods described by Hwang et al. (2010). PKC protein was hydrolyzed under optimal conditions by papain at a ratio of 50:1 in a phosphate buffer solution (50 mM), pH 6.5, at 65 °C using a water bath shaker at an agitation rate of 150 rpm for 6 h. The enzyme was inactivated by heating the sample at 100 °C for 10 min. The papain generated protein hydrolysate was then fractionated using a reversed-phase HPLC (1200 series, Agilent Technologies) on a semi-prep ZORBAX 300SB C18 column (9.4×250 mm, 5 μm; Agilent Technologies, Santa Clara, CA, USA). The protein hydrolysate was further fractionated based on the isoelectric focusing electrophoresis using an OFFGEL system (Agilent Technology, Waldbronn, Baden-Wuerttemberg, Germany). The fraction with higher ACE inhibitory activity was selected to identify the peptide sequences using tandem mass spectrometry. Peptides were analyzed using an Ultra-High Performance Liquid Chromatography system (1290 UHPLC, Agilent Technologies, Santa Clara) coupled with a high-resolution, accurate mass hybrid quadrupole-time of flight (Q-TOF) mass spectrometer (6540 Q-TOF, Agilent Technologies, Santa Clara). After peptide identification, the peptide sequences were synthesized by 1st BASE company, (JTC MedTech Hub, Singapore) and used for further studies.

### 2.3 Angiotensin-converting enzyme (ACE) inhibitory activity evaluation

ACE inhibitory potency of peptides was determined following the method described by Forghani et al. (2016). A peptide solution (15 μL) was incubated with ACE (10 μL, 100 mU/mL) at 37 °C for 10 min. The mixture was incubated with Hippuryl-histidyl-leucine (HHL) solution (50 μL) containing 0.1 M borate buffer pH 8.3 and 0.3 M NaCl at 37 °C for 60 min. To terminate the enzymatic reaction, 75 μL of 1 M HCl was added to the mixture. Subsequently 150 μL of pyridine were layered followed by addition of 75 μL of benzenesulfonyl chloride, and the mixture was vortexed for 1 min before being cooled on ice. The absorbance was recorded at 410 nm using a micro plate reader (Labomed, model UVD-2950, USA Power Wave X 340, Biotek Instruments. Inc.,Winooski, VT, USA). The standard curve of hippuric acid (HA) was constructed by plotting absorbance versus _mol of HA produced in 1 mL of reaction volume.

### 2.4 Effect of ACE on papain-generated peptides activity

The effect of ACE on papain-generated peptides was analyzed following the method of Yang et al. (2003) with some modifications. Briefly, 225 µL of the peptide solution of YGIKVGYAIP,

GGIF, GIFE at concentrations of 2, 4, and 4 mM were separately incubated with 150 µL of ACE (100 mU/mL) at 37 °C. Samples were withdrawn at incubation times of 0, 0.5, 1, 2, and 3 h and kept on ice prior to analysis by HPLC. A 20-µl sample was loaded onto a Hypersil GOLD C_18_ column (4.6×250 mm) attached to a Shimadzu (Kyoto, Japan) LC 20AT apparatus. The column was conditioned with eluent A (0.1 % TFA in DI) and eluted with 100 % eluent A from 0 to 10 min. The peptides were eluted with eluent B (0.1 % TFA in CH_3_CN) with a gradient elution of 0–100 % for 10–50 min. The absorbance was read at 215 nm.

### 2.5 Kinetics study of ACE inhibition

To determine the mode of enzyme inhibition, Lineweaver-Burk plots of 1/v versus 1/[S] were used. ACE inhibition was determined in the presence of five concentrations of HHL from 15 to 4000 µM and four concentrations of peptides, as shown in Table 1.

**Table 1:** Peptide concentrations used for kinetic study.

	Peptides	Peptide concentration (µM)			
1	YGIKVGYAIP	125	62	31	15
2	GGIF	2000	500	250	31
3	GIFE	4000	2000	125	16

Enzyme activity was expressed as nmol hippuric acid produced per min of enzymatic reaction. *K*_iu_ was calculated from the plot of 1/V_max _against inhibitor concentration at the intercept on the inhibitor concentration axis (Cornish-Bowden, 2013). *V*_max_ and *K*_m_ were calculated using GRAPHPAD PRISM 5 (GraphPad, Software Inc., San Diego, CA). Michaelis-Menten and Lineweaver-Burk plots were also created using the same software.

### 2.6 Molecular docking studies

Molecular docking was conducted using Glide (Glide, version 6.7, Schrödinger, LLC, New York, NY, 2015). The structures of YGIKVGYAIP, GGIF and GIFE were generated with Molecular Operating Environment software (MOE 2014.0901, Chemical Computing Group, Inc., Montreal, QC, Canada, 2014), and the energies were minimized using the MMFF94 program. For ACE docking, a crystal structure of human ACE bound to Captopril as the ACE-inhibitory drug (PDB ID 1UZF) was employed as an angiotensin converting enzyme (ACE) molecule. A binding site was generated by digitally removing captopril with a radius of 20 Å, with the coordinates ×  = 36.98, y = 27.05, and z = 50.65. Glide extra precision (XP) docking was performed for all ACE peptides and captopril as benchmarked. All water molecules in the structure of protein were digitally detached, and hydrogen atoms were added to the structure of protein. Binding energy values and the scores were used to evaluate the molecular docking to determine the best poses for the peptides. The binding affinity of PBP and the ligand complex was reported in the Glide scores (kcal/mol). Glide scores (kcal/mol) are shown in negative values, and those positions with a more negative value exhibit a potent interaction between protein and ligand. The Glide pose viewer was employed to analyze the resulting docked poses and to detect the hydrogen bonds and the hydrophobic, hydrophilic, electrostatic, and coordination interactions of ACE and peptides. The best docking poses with low Glide scores and the least binding energy were subjected to further analysis.

### 2.7 Statistical analysis

The results are presented as the mean ± standard deviation derived from three replications. The statistical analysis was carried out using Minitab 16.0 software (MINITAB, State College, PA, USA). Analysis was carried out using a one-way analysis of variance (ANOVA). A difference at p < 0.05 was taken to be statistically significant.

## 3 Results and discussion

### 3.1 Effect of the catalytic activity of angiotensin converting enzyme on bioactive peptides

To evaluate the possible degrading effects of ACE on the bioactive peptides generated from palm kernel cake proteins prior to examining their inhibitory activity, the peptides were preincubated with ACE in a water bath shaker at 37 ˚C for 0.5 h, 1 h, 2 h and 3 h, and the resulting peptides were analyzed using RP-HPLC.

**Fig. 1:**
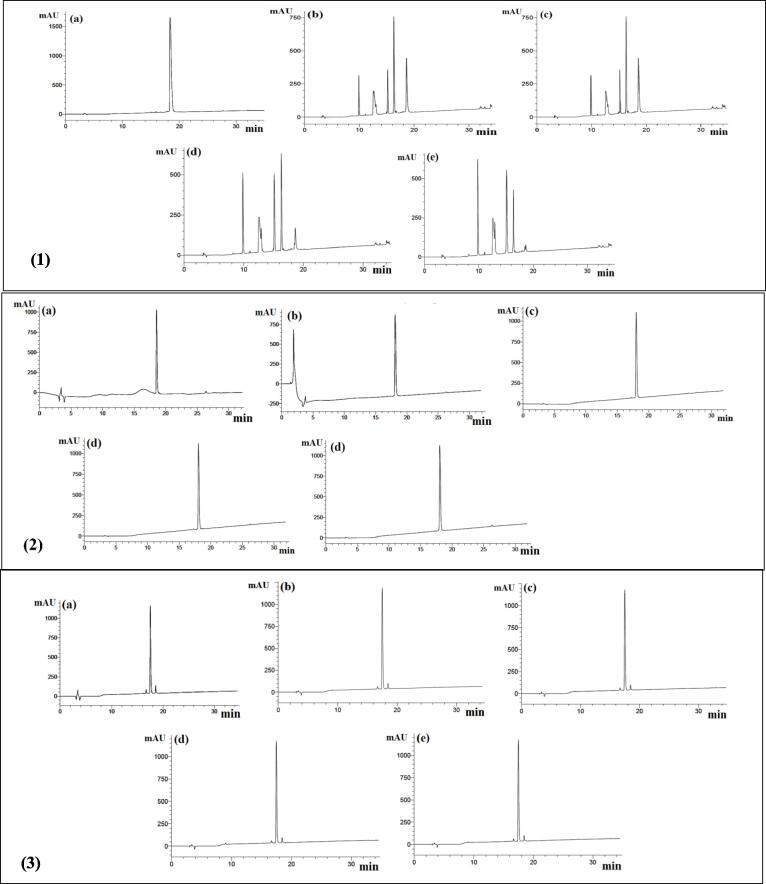
**RP-HPLC chromatograms of hydrolysis of peptides by ACE.** (1) YGIKVGYAIP; (2) GGIF; (3) GIFE; (a) before incubation; (b) 0.5 h of incubation; (c) 1 h of incubation; (d) 2 h of incubation; (e) 3 h of incubation. 20 μL of sample was eluted by mobile phase A (0.1 % of TFA in deionized water) and 0–60 % gradient of mobile phase B (0.1 % of TFA in CH_3_CN) for 5–35 min on a C_18_ column.

The RP-HPLC chromatograms of the peptides YGIKVGYAIP, GGIF and GIFE, preincubated with ACE, are shown in Fig. 1. The number of visible peaks for peptide YGIKVGYAIP increased to five peaks after 3 h of pre-incubation. The degree of degradation of the peptide by ACE was 70 % after the first 30 min of total pre-incubation time (Table 2). Although it has been suggested that peptides that contain proline are more resistant to degradation by degrading enzymes such as ACE (Vermeirssen et al., 2004), the percentage of the peptide cleavage increased to 98.5 % at the third hour of pre-incubation time, while at 2 h pre-incubation time, the peptide was degraded by 95 %. This suggests that peptide YGIKVGYAIP was not resistant to ACE, and it was probably degraded to YG, IK, VG, YA and IP, according to the previous studies showing that ACE can degrade a substrate at positions of I, V (Yang et al., 2003) and Y (Fujita & Yoshikawa, 1999). Concomitantly, the ACE inhibitory activity of YGIKVGYAIP decreased from 100 % to 93 % after pre-incubation (Table 3).

**Table 2:** Hydrolysis of papain-generated peptides by ACE with incubation for 3 h.

Bioactive peptides						
Incubation time (h)	YGIKVGYAIP		GGIF		GIFE	
	Peaks	Cleavage (%)	Peaks	Cleavage (%)	Peaks	Cleavage (%)
0.5	5	70 ± 2.2	1	1 ± 0.1	1	0 ± 0.3
1	5	84 ± 1.6	1	1 ± 0.2	1	0 ± 0.1
2	5	95 ± 3.2	1	1 ± 0.4	1	0 ± 0.1
3	5	98 ± 3.1	1	2 ± 0.6	1	0 ± 0.2

**Table 3:** ACE-inhibitory capacities (%) of peptides with and without pre-incubation with ACE.

Peptide number	ACE-inhibitory capacity (%)		Classification
	Without pre-incubation	With pre-incubation	
YGIKVGYAIP	100 ± 3.00	93 ± 2.40	Substrate type*
GGIF	97 ± 2.18	87 ± 2.70	Substrate Type**
GIFE	40 ± 1.00	43 ± 1.80	True inhibitor

* Pro-drugs are substrates for ACE which they are converted to true inhibitors by ACE.

** True inhibitors are substrates for ACE, which they are converted to inactive peptides, and pro-drug peptides.

Peptide GGIF was resistant to ACE, as the number of peaks did not increase after 3 h of pre-incubation (Fig. 1.3). The percentage of peptide cleavage was constant at approximately 1.5 % during the incubation period, suggesting that peptide GGIF was not degraded much by ACE (Table 2).

Fig. 1.4 shows peptide GIFE before and after pre-incubation with ACE. As shown, ACE had no effect on the structure of peptide GIFE, as the number of peaks produced with and without pre-incubation did not increase (Table 2). The chromatograms of the peptide showed one peak without pre-incubation, and the same peak could also be seen after the third hour of pre-incubation. The percentage of peptide cleavage by ACE was almost 0 %. The ACE inhibitory activity of the peptide (GIFE) was not significantly different before or after pre-incubation with ACE, at 43 % and 40 %, respectively (Table 3), indicating that this peptide is a true-inhibitor type. ACE inhibitory peptides can show their activities in three ways, so they are classified as an inhibitor-type, substrate-type, and pro-drug type based on changes in ACE inhibitory activity after hydrolysis of peptides by ACE (Fujita & Yoshikawa, 1999). Inhibitor-type peptides are ACE inhibitory peptides whose activity is not significantly altered as the peptides are resistant to cleavage by ACE. Substrate-type ACE inhibitors show a decrease in ACE activity due to cleavage by ACE in which peptides YGIKVGYAIP and GGIF could be categorized in this group. Pro-drug type refers to the conversion to potent ACE inhibitors following hydrolysis of larger peptide fragments by ACE itself.

As shown in Figs. 1.3 and 1.4, peptides GGIF and GIFE have Gly, Ilu, and Phe residues in their sequences. However, two of their residues (Gly and Glu) are not hydrophobic. Moreover, has ACE did not degraded both peptides after pre-incubation for 3 h. This is likely due to the weaker binding of the peptides to the active site of ACE compared to more hydrophobic peptides.

**Fig. 2:**
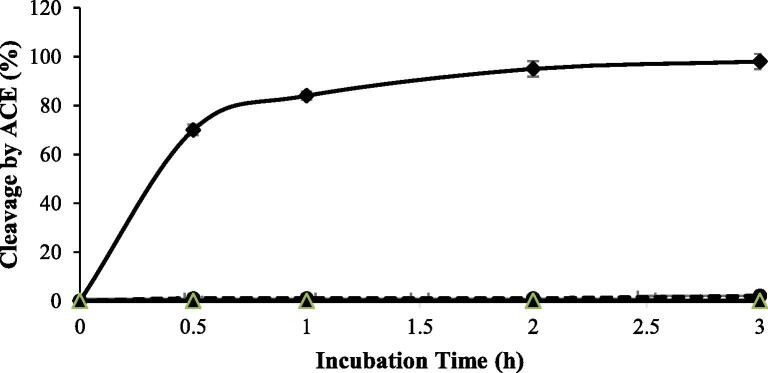
Time course of cleavage of peptides bonds by ACE.

Fig. 2 shows the time course of cleavage (%) of peptide bonds with ACE. As shown, peptide YGIKVGYAIP exhibited significant cleavage (%) by ACE in the first 30 min of incubation time, while peptides GGIF and GIFE did not show significant cleavages (%) over the incubation period, suggesting that GGIF and GIFE were more resistant than the other peptide. The cleavage percentage of the peptide YGIKVGYAIP reached a plateau after 30 min of incubation time.

ACE inhibitory peptides are categorized into three classes based on their interactions with ACE: (Fujita et al., 1995) true inhibitors, for which ACE inhibitory activity is not affected by pre-incubation with ACE; substrate type inhibitors, which function as a substrate for ACE that can be converted into inactive peptides with weaker ACE inhibitory activity; and finally, a pro-drug peptide, which is also a substrate for ACE but can be converted by ACE into a true inhibitor. It has been reported previously that only true inhibitors and pro-drugs can reduce blood pressure (Fujita et al., 2000, Yokoyama et al., 1992).

It should be noted that the ACE-inhibitory peptides GIFE and GGIF were categorized as true inhibitors, as their ACE inhibitory activity did not change significantly during pre-incubation. Although, the ACE inhibitory activity of peptide YGIKVGYAIP decreased after pre-incubation with ACE but it was not significant and it showed the lowest IC_50_, thus, it can be classified as the prodrug type.

### 3.2 Inhibition kinetics of bioactive peptides toward ACE

**Fig. 3:**
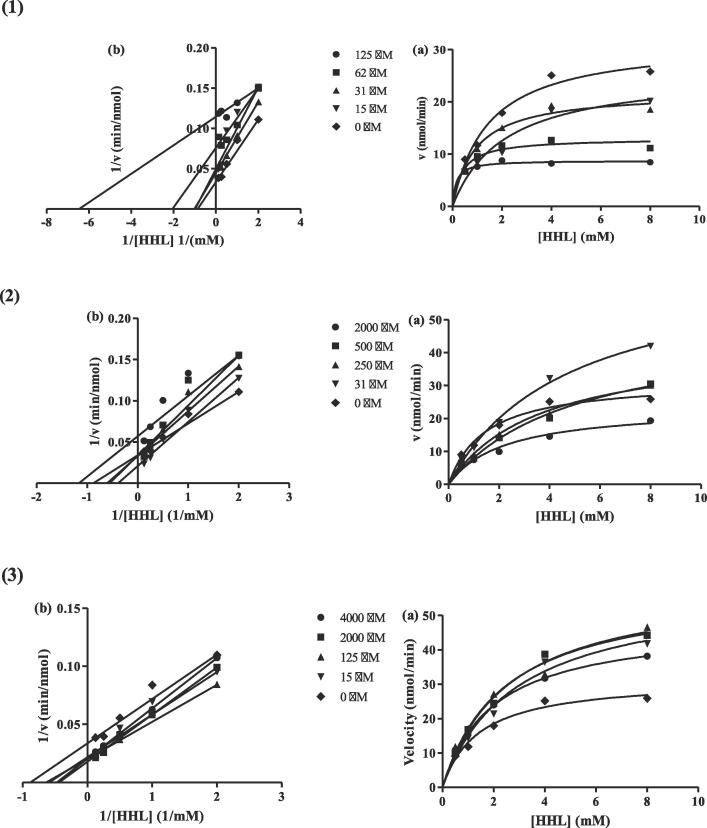
**Michaelis*-*Menten (a) and Lineweaver**-**Burk plot (b) of peptides.** Each point represents the mean of three experiments. ACE activities measured in the absence or presence of peptides. (1) YGIKVGYAIP; (2) GGIF; (3) GIFE.

To determine the inhibition kinetics of the ACE-inhibitory peptides YGIKVGYAIP, GGIF and GIFE, generated from palm kernel cake proteins, Lineweaver-Burk and Michaelis-Menten plots were constructed, as shown in Fig. 3. The calculated K_m_ (Michaelis constant)_,_ V_max_ (maximum reaction velocity), CE (catalytic efficiency) and K_iu_ (enzyme-inhibitor dissociation constant) values for the bioactive peptides are summarized in Table 4.

**Table 4:** *V*_max_, *K*_m_ of ACE inhibited by peptides along with its *K*_iu_ and CE.

Sequence/Peptide number	Peptide concentration (µM)	*K*_m_ (mM)	*V*_max_ (nmol/min)	$K_{m}^{app}$(mM)	$V_{max}^{app}$(nmol/min)	*CE (*$V_{max}^{app}$*/*$K_{m}^{app}$*)*	*K*_iu_ (mM)
YGIKVGYAIP	125 62.0 31.0 15.0			0.1465 0.3881 0.9271 2.1330	8.780 13.01 21.94 25.90	59.93 33.52 23.66 12.14	0.054
Control	0.00	1.451	31.66			21.82	
GGIF	2000 500 250 31.0			2.219 5.006 3.602 4.724	23.71 48.58 42.85 67.46	10.68 9.70 11.89 14.28	1.27
Control	0.00	1.450	31.74			21.88	
GIFE	4000 2000 125 16.0			2.007 2.559 2.420 2.871	47.79 59.71 58.29 58.06	23.81 23.33 24.08 20.22	18
Control	0.00	1.420	31.70			22.32	

The K_m_ values for ACE inhibitory activity in the absence of peptides YGIKVGYAIP, GGIF and GIFE were approximately 1.4 mM, while those in the presence of peptides YGIKVGYAIP, GGIF and GIFE at concentrations of 15 to 125 mM, 31 to 2000 mM and 16 to 4000 mM were calculated as 0.1465–2.1330 mM, 2.219–4.724 mM, and 2.007–2.871 mM, respectively. For peptide YGIKVGYAIP, the K_m_ value of ACE in the absence of the peptide was obviously higher than the $K_{m}^{app}$ values of ACE in the presence of the peptide at various concentrations, except for a peptide concentration of 15 µM, while peptides GGIF and GIFE showed lower K_m_ values in the absence of peptide, implying that the ACE reaction requires more substrate for catalysis in the presence of peptides GGIF and GIFE at all concentrations and YGIKVGYAIP only at a concentration of 15 µM, so that the efficiency of ACE was lower for GGIF and GIFE compared to ACE in the absence of peptides. In the presence of peptides YGIKVGYAIP, GGIF and GIFE, the calculated $K_{m}^{app}$ increased by decreasing the peptide concentration (Table 4), implying that at low peptide concentration, more substrate was required for enzyme catalysis. Hence, ACE at a lower concentration of peptide was less effective than at a higher peptide concentration. Moreover, at a high concentration of peptide, more peptides are bound to the active site and prevent the formation of an enzyme-substrate complex. This result is in accordance with that reported by Girgih, He & Aluko (2014). Furthermore, kinetic studies showed that the inhibition mode of the peptides at different concentrations show mixed-type inhibition because the x-axis and slope lines do not intersect at the same point and the K_m_ values of the control and various peptide concentrations were not the same. This demonstrates that the peptides can compete with the substrate to bind to the active site of the enzyme and at other non-active sites.

As shown in Table 4, the V_max_ values of the uninhibited ACE reaction for peptides YGIKVGYAIP, GGIF and GIFE were 31.66 nmol/min, 31.74 nmol/min and 31.70 nmol/min, respectively. The $V_{max}^{app}$ of the ACE reaction in the presence of peptides was reduced with increasing peptide concentrations in all three assayed peptides. However, the V_max_ of YGIKVGYAIP was significantly lower than GGIF and GIFE at all peptide concentrations, suggesting that YGIKVGYAIP was more effective, consistent with its IC_50_ value (IC_50_ = 1 µM). The catalytic efficiency (CE) of the uninhibited ACE reaction was 21.82, 21.88 and 22.32, which increased with increasing peptide concentration. Among the different peptide concentrations, the peptide concentration of YGIKVGYAI at 15 µM and GGIF at 500 µM showed the lowest CE (12.14 and 9.70, respectively), indicating that enzyme catalysis decreased at these peptide concentrations so that the inhibition mode increased. In addition, the catalytic efficiency of ACE in the absence of the peptides YGIKVGYAI and GIFE was lower than the CE in the presence of higher concentrations of these two peptides, implying that ACE efficiently converts the substrate to product, even in the presence of peptides. CE measures how efficiently the enzyme converts the substrate (the molecule it acts on) to the product. Peptide YGIKVGYAIP showed the lowest K_iu_ of 0.054 mM among the peptides in this study. This value is consistent with the IC_50_ value obtained for this peptide, which was the lowest value among the peptides generated from PKC. The K_iu_ value obtained for peptide YGIKVGYAIP is lower than the K_iu_ value of RALP (0.1041 mM) and TF (12.2726 mM) and is higher than the 0.0312 mM previously reported by He, Aluko & Ju (2014) for the peptide.

Moreover, the Kiu value of this peptide is lower than peptides WVYY (0.06 mM) and WYT (1.83 mM) reported by Girgih, He & Aluko (2014). Peptide GIFE showed the highest K_iu_ (K_iu_ = 18) among other peptides, indicating that this peptide has the lowest affinity to ACE. This peptide also showed the lowest ACE inhibitory activity (40 %) among the peptides.

### 3.3 Molecular docking studies

**Fig. 4:**
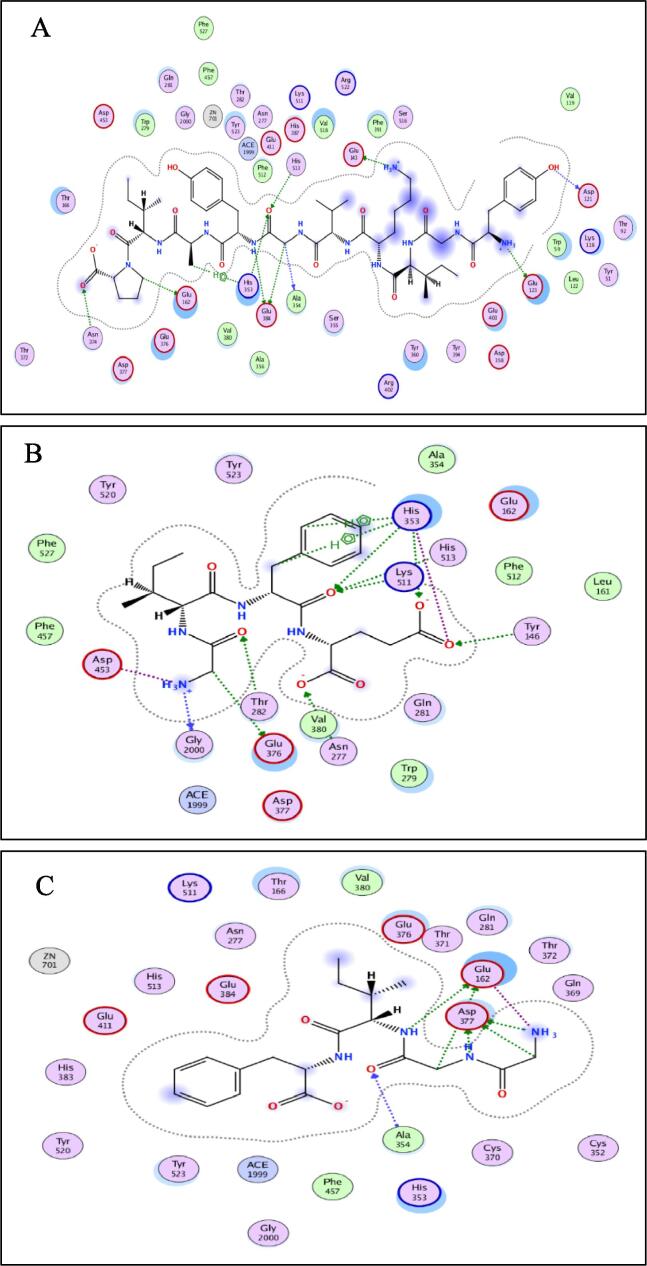
Automated molecular docking of YGIKVGYAIP (A), GGIF (B), and GIFE (C) at the angiotensin-converting enzyme (ACE) active site. ACE hydrophobic residues are represented in green, positively charged residues in blue, and negatively charged residues in red and hydrogen bonds as purple arrow, polar residues in turquoise color, while other residues and the zinc atom are represented automatically. Image obtained with Accelrys DS Visualizer software. (For interpretation of the references to color in this figure legend, the reader is referred to the web version of this article.)

Fig. 4 indicates the presence of the best poses for YGIKVGYAIP, GGIF and GIFE within the catalytic site of ACE. The stability of the best pose for each peptide was accomplished through hydrogen bonding and electrostatic and hydrophobic interactions between amino acid residues of the ACE binding domain and those of the individual peptide within a distance of 3.5 Å. YGIKVGYAIP exhibited higher number of total interactions (21) with ACE, which could be the main reason for the higher ACE inhibitory activity (lower IC_50_ value of 1 µM) compared to GGIF and GIFE, which indicated 15 and 15 interactions, respectively. This was ascertained by the lowest electrostatic binding energy of −140.003 kJ/mol for YGIKVGYAIP compared to −118.305 kJ/mol for GGIF and −131.691 kJ/mol for GIFE, as shown in Table 5. Similarly, the van der Waals energy of −6.6593 kJ/mol was lower for YGIKVGYAIP compared to GGIF (−3.637 kJ/mol) and GIFE (−5.7135 kJ/mol). As shown in Table 4, the binding energy data revealed the higher ACE-binding affinity (lower Ki value of 0.054) of YGIKVGYAIP compared to GGIF and GIFE, respectively. The higher activity of YGIKVGYAIP may also be due to a higher degree of interactions with the active site of ACE. As shown in Table 5, it appears that the ability of peptides to develop numerous hydrogen bond interactions with ACE may be a major factor in the ACE inhibitory activity of the peptide and stabilization of the ACE-peptide complex structure.

**Table 5:** ACE inhibitory activities and docking study characteristics of peptides.

Title	Peptide sequence	ACE inhibitory activity (%)	ACE inhibitory activity (IC_50_, µM)	Electrostatic interaction (kJ/mol)	Hydrophobic interaction (kJ/mol)	Van der Waals (kJ/mol)	Docking Score
1	YGIKVGYAIP	100 ± 3.00	1.0	−140.003	−2.005	−6.659	−14.733
2	GGIF	97 ± 2.17	92	−118.305	−0.827	−3.637	−8.006
3	GIFE	40 ± 1.00	3.0	−131.691	−1.398	−5.713	−13.800

A similar pattern of hydrogen bonds with ACE residues has been reported for peptides WVYY (7 bonds) and WYT (5 bonds) (Girgih et al., 2014). Furthermore, the IC_50_ is correlated to the number of hydrogen bonds formed. As shown in Table 5, YGIKVGYAIP revealed a lowest IC_50_ of 1 µM and formed 12H-bonds, followed by GIFE, with an IC_50_ of 3 µM and GGIF with 92 µM. Thus, it appears that the number of hydrogen bonds and ACE residues involved play a prominent role in the ACE-inhibitory capacity of the peptides. This is because the more potent YGIKVGYAIP formed one H-bond with His353 and one with His513, but no H-bond with Gln281(Table 6); all of these amino acid residues are important constituents of the ACE active site. GIFE showed one H-bond with His513 and four H-bonds with His353, but its total H-bond number was 11, which is lower than that of YGIKVGYAIP (12). The weaker-acting GGIF does not form any H-bonds with Gln281, His353, and His513.

**Table 6:** Residues of tACE with at least one atom at a distance of 3.5 Å near a docked peptide

tACE residues	YGIKVGYAIP	GGIF	GIFE
Trp59			
Asn66			
Asp121	1 H-donor		
Glu123	2 H-donor, 2 Ionic		
Arg124			
Glu143	1 H-donor, 1 Ionic		
Tyr146			1 H-acceptor
Glu162	1 H-donor	2 H-donor, 1 Ionic	
Thr166			
Met223			
Asn277			1 H-acceptor
Gln281			
Thr282			1 H-acceptor
His353	1 H-acceptor		2 H-acceptor, 2 Ionic, 2 H-pi
Ala354	1 H-donor	1 H-acceptor	
Ala356			
Asp358			
Cys370			
Thr372			
Asn374	1 H-acceptor, 1 H-pi		
Glu376			1 H-donor
Asp377		3 H-donor, 2 Ionic	
His383	1 Metal	1 Metal	
Glu384	2 H-donor		
His387	1 Ionic, 1 Metal	1 Ionic, 1 Metal	
Phe391			
Glu403			
His410			
Glu411	2 Ionic, 1 Metal	2 Ionic, 1 Metal	
Phe457			
Asp453			1 Ionic
Lys511			1 H-acceptor
Phe512			
His513	1 H-acceptor		1 H-acceptor
Ser516			
Tyr520			
Arg522			
Tyr523			
Phe527			
ACE1999			
Gly2000			1 Ionic, 1 H-donor
ZNB701			
**Total H-bond**	**12**	**6**	**11**
**Total Interaction**	**21**	**15**	**15**

## 4 Conclusions

Interaction of ACE with three novel papain-generated bioactive peptides from palm kernel cake proteins namely YGIKVGYAIP, GGIF and GIFE was studied by molecular docking simulation. Furthermore, the inhibition kinetics and the stability of the peptides against ACE were studied. Lineweaver-Burk plots revealed that the inhibition mode of the peptides at different concentrations was mixed-type inhibition, implying that the peptides can compete with the substrate to bind to the active site of the enzyme and at other non-active sites. Moreover, study of peptide after incubation with ACE showed that peptide YGIKVGYAIP was degraded, while peptides GGIF and GIFE were resistant and were not degraded. Additionally, results exhibited that YGIKVGYAIP and GGIF were substrate-type whereas GIFE was a true-inhibitor type. Moreover, based on molecular docking study, YGIKVGYAIP exhibited higher number of total interactions in comparing to peptides GGIF and GIFE which could be a reason for lower IC_50_ of YGIKVGYAIP.

## Declaration of Competing Interest

The authors declare that they have no known competing financial interests or personal relationships that could have appeared to influence the work reported in this paper.

## Data Availability

No data was used for the research described in the article.
